# Advances in Semantic-Preserving Text Watermarking

**DOI:** 10.3390/s26051528

**Published:** 2026-02-28

**Authors:** Jiale Meng, Zheming Lu

**Affiliations:** School of Aeronautics and Astronautics, Zhejiang University, Hangzhou 310027, China; mengjiale@zju.edu.cn

**Keywords:** font-based watermarking, format-based watermarking, image-based watermarking, semantic-preserving, text watermarking, document security, survey

## Abstract

Textual content faces escalating security threats regarding copyright infringement, tampering, and unauthorized distribution. Text watermarking offers a vital defense mechanism by embedding imperceptible identifiers for source tracking and anti-counterfeiting. However, unlike general image watermarking, protecting text is uniquely challenging due to its highly discrete structure and low pixel redundancy, where even minute perturbations can compromise legibility. Over the past three decades, a wide range of text watermarking techniques have been proposed to address these challenges. While recent research has heavily favored semantic-based watermarking driven by Large Language Models (LLMs), these approaches are often inapplicable to high-stakes scenarios requiring strict content integrity and visual fidelity, such as legal documentation and artistic font protection. Addressing this gap, this paper presents a comprehensive survey of semantic-preserving text watermarking methods developed in recent years, with a particular focus on image-based, font-based, and format-based techniques. We propose a unified classification framework to systematically analyze these approaches, examining their methodological principles, robustness, embedding capacity, and imperceptibility. By clarifying the core characteristics and limitations of existing techniques, this survey aims to provide a structured technical reference for researchers and practitioners, facilitating the advancement of secure, robust, and scalable text protection technologies.

## 1. Introduction

In the era of information digitization and global interconnectivity, text plays a pivotal role in both daily life and professional domains. From legal documents, commercial contracts, and academic reports to personal signatures and customized font designs, textual content is ubiquitous across various digital and physical media, carrying significant legal efficacy, commercial value, and cultural significance.

However, the rapid advancement of internet technology and digital dissemination has made the replication and distribution of textual information extremely convenient. While this enhances information accessibility, it simultaneously introduces unprecedented security risks. For instance, confidential documents are frequently printed and distributed offline to evade digital tracking, and electronic signatures are often extracted to forge critical documents. These phenomena pose severe challenges to the intellectual property rights and authenticity of textual content. Consequently, there is an urgent need for a technological solution capable of providing source tracking, anti-counterfeiting, and copyright protection without compromising the readability or visual integrity of text. Text watermarking technology has emerged in this context, offering a novel approach to ensuring the security and trustworthiness of textual content. This constitutes the primary focus and subject of in-depth discussion in this survey.

In general, *text watermarking* aims to embed imperceptible and unique identification information into textual content to achieve objectives such as copyright protection, tamper detection, and source tracking. Although image watermarking techniques have achieved significant success in the visual media domain [1,2,3], these methods are difficult to apply directly to textual scenarios. The fundamental reason is that image watermarking is mostly designed for grayscale or color images, which possess rich pixel distributions and have substantial redundancy available for watermark embedding. In contrast, text images exhibit a highly discrete structure characterized by limited pixel redundancy, typically consisting of only binary (black and white) pixels. Consequently, any minute pixel perturbation in a text image may lead to noticeable changes in character morphology or readability, making it easily perceptible to the human eye. Therefore, embedding watermarks into textual content requires specialized designs that address its discrete structure and low-redundancy characteristics.

### 1.1. What Is Semantic-Preserving Text Watermarking?

Current text watermarking methods can be broadly categorized into semantic-based (or linguistic-based) methods and semantic-preserving methods. The former encodes watermark information by exploiting the degrees of freedom in syntactic structures while preserving textual semantics, such as syntactic transformations and constituent reordering; the latter does not directly modify the textual content itself, but instead embeds watermarks by manipulating the visual presentation or layout attributes of the text. Semantic-preserving text watermarking methods can be broadly categorized into three classes: image-based methods, font-based methods, and format-based methods. Image-based methods operate via pixel- or feature-level perturbations, font-based methods encode information through glyph modulation, and format-based methods rely on subtle variations in layout and formatting attributes.

### 1.2. Why a Survey for Semantic-Preserving Text Watermarking?

In recent years, driven by the rapid development of Large Language Models (LLMs), research on semantic-based text watermarking has been relatively abundant [4,5,6,7,8]. However, semantic-based methods [9,10,11,12] suffer from inherent limitations that restrict their applicability in many practical scenarios. **First**, they primarily manipulate linguistic meaning while largely ignoring visual presentation, which makes them unsuitable for applications where visual characteristics are essential, such as handwritten text, artistic fonts, or personalized typographic designs. **Second**, in high-sensitivity documents, including legal texts, official correspondence, and contracts, content fidelity is critical, and any modification to the textual content is typically unacceptable, rendering semantic alteration strategies impractical. **Third**, when text exists only in visual form, such as scanned documents, signature images, or embedded-font images, semantic-level methods cannot be directly applied for watermark embedding or extraction. In contrast, image-based, font-based, and format-based watermarking techniques achieve robust embedding and extraction by manipulating visual appearance or layout attributes while maintaining both semantic integrity and visual consistency. As a result, they exhibit clear advantages in scenarios where semantic modification is infeasible or undesirable.

However, despite their practical relevance, existing studies on these semantic-preserving approaches remain fragmented. Systematic surveys that provide a unified classification framework and a comprehensive comparative analysis across these categories are still scarce. To the best of our knowledge, no dedicated survey focusing on image-based, font-based, and format-based text watermarking methods has been published in the past five years.

In this paper, we present a systematic review of non-semantic text watermarking methods. We comprehensively categorize and analyze image-based, font-based, and format-based approaches from multiple perspectives, including methodological principles, robustness, embedding capacity, and imperceptibility. Compared to previous reviews such as [13], which primarily categorized text watermarking into format-based, linguistic-based, and image-based methods, this survey provides a more granular and updated landscape. Specifically, we elevate font-based watermarking to a primary research pillar, reflecting its rapid evolution from manual glyph adjustments to deep-generative vector font synthesis. Furthermore, while earlier studies were limited to pre-2019 literature, our work bridges a critical five-year gap by analyzing state-of-the-art breakthroughs up to 2025.

### 1.3. How the Related Works Are Collected?

We conducted a systematic search across databases, including IEEE Xplore, ACM Digital Library, and Google Scholar. The search spanned from early foundational works (1994) to the latest advances in 2025. Key terms included ‘text watermarking’, ‘font watermarking’, and ‘document watermarking’. Inclusion criteria focused on semantic-preserving methods, excluding purely linguistic/LLM approaches unless used for comparison.

The remainder of this paper is organized as follows: Section 2 introduces the basic definitions and key algorithm attributes of text watermarking, laying the foundation for subsequent chapters. Section 3, Section 4 and Section 5 review the three main categories of text watermarking methods, which are image-based, font-based, and format-based techniques, respectively. Section 6 explores the major challenges and potential research directions in the field. Finally, Section 7 concludes the paper.

## 2. Preliminaries of Text Watermarking

### 2.1. Text Watermarking Algorithms

A text watermarking framework generally consists of two principal components: a watermark embedder E and a watermark extractor R. Given an original text *X* and a watermark message *m*, the embedder generates a watermarked text *T* according to

(1) E(X,m)=T.

Semantic-based watermarking modifies the lexical or syntactic structure of the text, which may introduce minor semantic variations. In contrast, semantic-preserving approaches (e.g., image-based, font-based, and format-based) alter only the visual or structural representation while strictly preserving the linguistic semantics. This semantic distinction can be expressed as:

(2) $$\mathrm{sem}\left(T\right)\left\{\begin{array}{cc} \approx \mathrm{sem}(X), & \mathrm{for}\;\mathrm{semantic-based}\;\mathrm{watermarking}\\ =\mathrm{sem}(X), & \mathrm{for}\;\mathrm{semantic-preserving}\;\mathrm{watermarking} \end{array}\right.$$

The watermark message *m* may be either a zero-bit watermark, which serves solely to indicate the presence of watermarking, or a multi-bit watermark that encodes explicit information. The amount of information contained in *m* is referred to as the watermark payload. The watermark extractor R operates on any text *T* and outputs its predicted watermark message, formulated as

(3) R(T)=m.

### 2.2. Fundamental Attributes of Text Watermarking Algorithms

To provide a deeper understanding of text watermarking algorithms, this section identifies four fundamental attributes that define an effective text watermarking algorithm: robustness, generalizability, invisibility, and watermark capacity.

#### 2.2.1. Robustness

**Figure 1:**
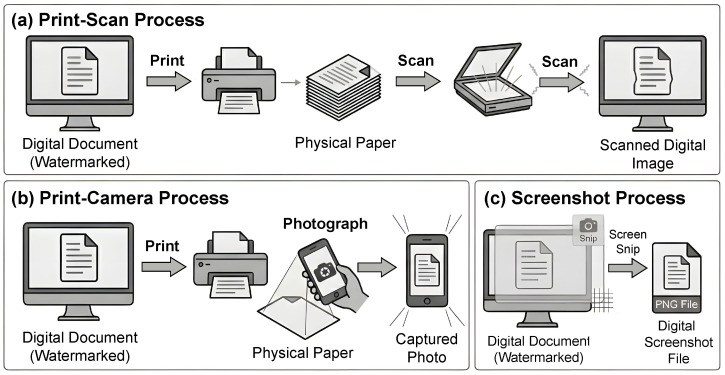
Illustration of the cross-media transmission process of watermarked documents.

During dissemination, text may be converted, printed, scanned, or photographed, all of which can degrade the embedded watermark signal, as shown in Figure 1. A robust algorithm ensures that the watermark remains detectable under such perturbations, thereby maintaining reliability as a proof of ownership or authenticity in practical scenarios.

#### 2.2.2. Generalizability

Generalizability refers to the adaptability of a watermarking algorithm across diverse languages (e.g., Chinese, English, and Arabic) and font styles without requiring specific re-tuning for each new case. For practical deployment in globalized office environments, an algorithm must maintain stable performance across various scripts and rendering engines to ensure its universal utility.

#### 2.2.3. Invisibility

Watermark embedding should introduce minimal perceptual distortion to the text. Using a text-quality evaluation function Ψ, invisibility is maintained if the quality deviation between the original text *X* and its watermarked version $E(X,m_{i})$ remains below a predefined tolerance θ; i.e., $\Psi (X,E\left(X,m_{i}\right))<\theta$.

#### 2.2.4. Watermark Capacity

Beyond ensuring security and imperceptibility, a watermarking system must provide adequate message-carrying capacity. An optimal scheme balances invisibility and robustness with the ability to encode substantial information, thereby enhancing its practical utility for applications such as ownership verification and multi-level authentication.

## 3. Image-Based Text Watermarking

**Figure 2:**
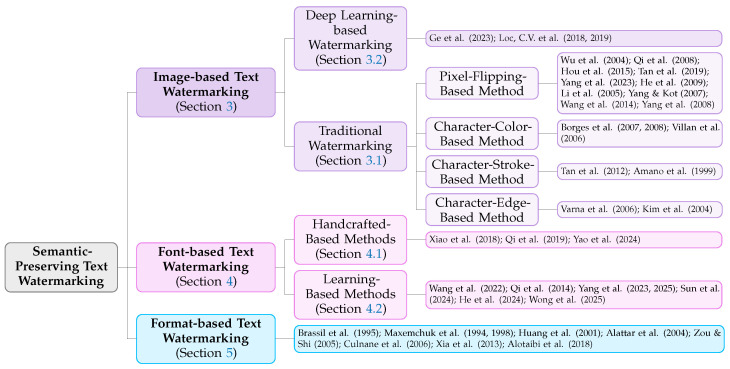
Taxonomy of text watermarking methods [14,15,16,17,18,19,20,21,22,23,24,25,26,27,28,29,30,31,32,33,34,35,36,37,38,39,40,41,42,43,44,45,46,47,48,49,50,51,52].

Image-based text watermarking aims to covertly and robustly embed secret information (i.e., the watermark) into text regions within an image. Its implementation mechanisms can be primarily categorized into two main approaches: traditional feature modulation methods and deep learning-driven methods, as shown in Figure 2. Traditional feature modulation methods encode information by applying minute statistical adjustments to the physical or geometric features of the text. This includes operations such as adjusting character brightness or color and flipping pixels in local regions of binary images. Deep learning-driven methods employ encoder–decoder networks to learn optimal embedding and extraction mappings in an end-to-end manner. During training, these methods simulate various attacks (e.g., print–scan, noise, and geometric transformations), enabling the network to automatically learn how to hide information within pixel residuals that are least sensitive to distortion. In the following sections, we will systematically review and analyze these two technical trajectories.

### 3.1. Traditional Feature Modulation Methods

Traditional feature modulation methods rely on an in-depth analysis of the structural characteristics of text images. They embed watermark information by applying minute modulations to specific features through carefully designed rules or algorithms. These methods typically possess strong interpretability and low computational complexity, retaining significant value in resource-constrained scenarios or contexts requiring explicit security analysis. Based on the different features modulated, traditional methods can be systematically classified into four main categories: pixel flipping-based, character color-based, character stroke-based, and character edge-based methods. The core ideas, representative works, and limitations of each category are detailed below.

#### 3.1.1. Pixel-Based Modulation

Pixel flipping methods [1,18,19,20,21,22,23,25,26,27,53,54] represent one of the earliest systematically studied technical routes in text image watermarking. The core idea is to identify “flippable pixels” in text images, defined as pixels whose reversal of the black/white state does not significantly affect character recognizability or visual quality. By selectively flipping these pixels, binary watermark information can be embedded. While this concept appears simple, it involves a profound understanding of the topological structure of binary images, human visual perception characteristics, and the physical properties of the printing and scanning process.

The pioneering work of Wu and Liu [18] systematically defined the concept of “flippable pixels” for the first time and established a comprehensive scoring metric to quantify pixel flippability. This metric incorporates two core factors: smoothness, which evaluates the impact of pixel flipping on local texture continuity by analyzing horizontal, vertical, and diagonal transitions within the pixel’s neighborhood, and connectivity, which ensures that flipping does not induce stroke disconnections or undesirable artifacts by counting the number of black and white connected components. Experimental results indicate that pixels located at character or stroke boundaries typically exhibit higher flippability scores. This is attributed to the fact that microscopic alterations at the boundaries naturally blend with inherent edge irregularities, rendering them imperceptible to the human visual system. Regarding the embedding strategy, Wu and Liu adopted a block-based encoding scheme. To address the uneven distribution of embedding capacity in binary images, the image is first subjected to a random permutation. The permuted image is then partitioned into blocks, where one bit of information is embedded by enforcing a specific statistical relationship (e.g., parity) among the flippable pixels within each block. Blocks lacking flippable pixels are automatically bypassed to preserve visual fidelity. However, this method suffers from a significant drawback: accurate extraction and synchronization between the encoder and the decoder necessitate the inclusion of visible registration marks. This requirement not only compromises the document’s aesthetic quality but may also reveal the existence of the watermark. Notwithstanding these limitations, Wu and Liu’s work established the foundational framework for flippable pixel watermarking, guiding research directions for the ensuing decade.

However, Wu et al. observed that their flipping strategy demonstrated limited robustness, exhibiting resilience only against high-quality print–scan processes. To overcome this vulnerability, subsequent research explored improvements in both the spatial and transform domains. Focusing on the spatial domain, Qi et al. introduced an innovative scheme founded on statistical invariants [19]. The core insight posits that the ratio of a character’s black pixel count (BPC) to the average BPC across all document characters remains essentially invariant after print–scan operations. This desirable property stems from the statistically similar degradation (i.e., blurring and noise) imposed on all characters by the print–scan channel. Building upon this observation, Qi et al. devised a sophisticated encoding mechanism: all characters are partitioned into an embedding region and an adjustment region. Watermark bits are carried by modulating the BPC of the embedding region via parity quantization. Simultaneously, the BPC of the adjustment region is modified to preserve the global BPC average, thereby realizing robust ratio-based encoding. The physical embedding is implemented using a boundary point flipping strategy: a calculated number of pixels along the character’s boundary are precisely selected and flipped according to the required BPC change. For extraction, the scheme is fully blind, requiring neither the original image nor registration markers. The process simply involves scanning the document, computing the character-to-average BPC ratio, and recovering the watermark bit via parity quantization. This method not only achieves excellent imperceptibility but also provides a practical capacity of one bit per character, significantly enhancing its utility.

Hou et al. [20] approached the problem from the perspective of multi-scale representation. The original binary image is decomposed into a series of multi-resolution thumbnails via perimeter expansion and systematic sampling. Watermark information is embedded by selectively flipping pixels within these thumbnails to modulate the BPC of specific regions, thereby establishing a predefined distribution pattern. This strategy, which encodes data into local density variations, demonstrates inherent robustness; since the print–scan process fundamentally functions as a low-pass filtering and resampling mechanism, it attenuates high-frequency details while preserving low-frequency statistical density features. During extraction, the scanned image is similarly decomposed into thumbnails, and the watermark is decoded by evaluating the deviation of the BPC of each thumbnail relative to the global average. Experimental results demonstrate that the proposed method exhibits significant robustness against print–copy–scan operations.

Of particular note is the approach proposed by Tan et al. [21], which leverages Fourier descriptors. While similarly exploiting the invariance of the character black pixel count ratio, this method employs a more sophisticated encoding mechanism: watermark information is embedded via quantization modulation of Fourier descriptors. This frequency domain representation offers superior resilience to the distortions induced by the print–scan process. Crucially, to guide the boundary pixel-flipping strategy, the method integrates constraints regarding character contour smoothness and connectivity. By employing an optimization algorithm, the scheme ensures that the modified characters not only accurately carry the watermark payload but also preserve their visual naturalness. Consequently, this meticulous control mechanism enables the method to achieve an optimal trade-off between robustness and imperceptibility.

Another significant contribution is the language–universal font watermarking scheme introduced by Yang et al. [22]. Central to this framework is centroid-shift encoding, where a specialized algorithm systematically modulates the boundaries or positions of character strokes to induce minute centroid displacements in predetermined directions. The watermark payload is embedded within the relative centroid positions of adjacent characters. By relying on intrinsic geometric relationships rather than absolute coordinates, this encoding strategy exhibits robust resilience against the geometric distortions and noise encountered during cross-media transmission. Crucially, the proposed centroid-shifting algorithm ensures that pixel modifications are executed in a systematic and coordinated manner. This approach avoids the visual artifacts often caused by random pixel flipping, thereby demonstrating superior applicability across diverse linguistic scripts.

Concurrently, Li et al. [53] proposed a robust document-watermarking algorithm based on the modification of stroke boundary pixels, effectively countering print–scan attacks. The core innovation of this approach lies in the segmentation of each text line into two sub-blocks; within these, the pixel row exhibiting the highest black pixel density is identified, and watermark bits are embedded by flipping the nearest boundary pixels of that specific row. The extraction process operates in a non-blind mode, necessitating the original PDF document as a reference. To mitigate distortions introduced by the print–scan channel, the scanned image undergoes preprocessing steps such as binarization and skew correction. Subsequently, an adaptive alignment model automatically calibrates the position of the watermarked image, allowing for information recovery through pixel-wise comparison. Experimental results demonstrate the method’s strong generalization capabilities across various Chinese fonts (e.g., SimSun, KaiTi, and FangSong) and English fonts (e.g., Arial and Times New Roman), with all Peak Signal-to-Noise Ratio (PSNR) values exceeding 32 dB, indicating superior imperceptibility. However, the method is constrained by its low embedding capacity and the requirement for the original text during extraction, which significantly limits its practical utility.

Recently, Meng et al. [1] proposed CoreMark, an image-based framework built upon a novel embedding paradigm termed CORE, which comprises several consecutively aligned black pixel segments. The pivotal advantage of CORE lies in its structural continuity, which effectively resists the discrete, random noise typical of print–scan processes. In the embedding phase, a dynamic module extracts COREs and conceals data by modulating their row counts, while referencing neighboring pixels to preserve character structure. Extraction is performed by comparing the width of each character’s CORE against an adaptive threshold. Furthermore, the study proposes a universal plug-and-play embedding strength regulator. By partitioning text lines into equal length sub-lines and embedding the identical bit across complete characters, this mechanism automatically increases the number of redundant characters as font size decreases, thereby adaptively enhancing robustness. Experimental evaluations encompassing various Chinese and English fonts, diverse font sizes, and six additional languages demonstrate that CoreMark achieves significant performance improvements against screen capture, print–scan, and print–camera attacks, achieving robustness, generalization, and imperceptibility simultaneously.

Distinct from purely spatial domain approaches, He et al. [23] pioneered a robust binary text watermarking algorithm based on the Discrete Cosine Transform (DCT), shifting the locus of robustness design to the transform domain. The core methodology involves transforming binary image blocks into the DCT domain and selecting a set of high-frequency coefficients via a threshold *K*. A seminal theoretical innovation of this approach is that watermark bits are encoded within the sign patterns of these coefficients rather than their magnitudes. This design is premised on the observation that while coefficient magnitudes fluctuate due to noise and blurring during the print–scan process, the signs of carefully selected high-frequency coefficients exhibit statistical stability. By utilizing the Inverse DCT (IDCT) to calculate necessary modifications, the algorithm precisely determines the set of pixels to be flipped in the spatial domain. This frequency-guided strategy ensures a uniform distribution of modifications, significantly minimizing perceptual impact while demonstrating superior robustness against print–scan and photocopying attacks.

As embedding capacity is a pivotal performance metric, significant research has focused on its optimization. Yang and Kot [25] mathematically revisited pixel flippability in 2007, proposing strict criteria based on local connectivity preservation. They evaluated three topological invariants within a 3×3 neighborhood: vertical–horizontal transition counts, internal right angles, and sharp angles. A pixel is deemed valid for flipping only if these metrics remain constant after modification. Comparative analyses against [18,54] demonstrated that this approach yielded superior visual quality while supporting a higher data payload. Subsequently, the authors established the theoretical equivalence between edge flipping and a one-unit edge shift in a binary image [27]. Leveraging this insight, they introduced the Interleaved Morphological Binary Wavelet Transform (IMBWT) to track and encode these microscopic shifts. The IMBWT’s interleaved decomposition facilitates blind extraction and increased capacity by 200–400% compared to their spatial domain counterpart [25], underscoring the advantage of wavelet domain representation. A further breakthrough in capacity was achieved by Wang et al. [26] through an innovative 7-ary encoding scheme. This method classifies 2×2 pixel blocks into specific patterns: non-symmetric blocks (NSB) and diagonal pair blocks (DPB), along with their complements. These form seven distinct categories (excluding uniform blocks), allowing each block to encode approximately $\log_{2}7\approx 2.8$ bits, nearly tripling the conventional one-bit limit. Empirical tests on standard images yielded capacities ranging from 29,148 to 53,229 bits, exceeding contemporary methods by one to two orders of magnitude. This work demonstrates that meticulously designed high-order encoding patterns can effectively transcend the capacity limitations of traditional binary pixel flipping. However, it must be noted that these high-capacity methods are primarily optimized for the digital domain, as their encoding relies on precise pixel-level registration. In the print-scan environment, geometric distortions (e.g., rotation, scaling, and nonlinear deformation) and noise destroy this exact spatial correspondence, resulting in a severe degradation of the methods’ robustness. Consequently, the inherent trade-off between embedding capacity and robustness continues to be the central challenge facing this class of watermarking schemes.

A comprehensive summary of the discussed pixel-based modulation methods is presented in Table 1. It compares the core embedding mechanisms of various approaches and assesses their overall performance in terms of imperceptibility, generalizability, and capacity, alongside their specific robustness to print-scan, print-camera, and screen capture.

#### 3.1.2. Color-Based Modulation

Unlike binary pixel flipping, color or grayscale text provides an additional embedding degree of freedom, as the character’s color or luminance can be continuously modulated within a defined range without significantly impairing readability. Methods based on character color [28,29,30] exploit this property by systematically modulating the color attributes of text elements to conceal the payload. This class of schemes is particularly applicable to high-quality printed materials, full-color documents, and electronic display environments.

The Text Luminance Modulation (TLM) scheme proposed by Borges et al. [28] serves as the foundational work in this domain. TLM begins by decomposing the document into fundamental text elements (e.g., characters, symbols, or lines). Watermark bits are embedded by systematically assigning a luminance gain to each element (e.g., increasing luminance for ‘1’ and decreasing or maintaining it for ‘0’). The modulation amplitude requires careful calibration: excessive amplitude impairs readability, while insufficient amplitude compromises reliable extraction after the print–scan process. The key innovation lies in the robust design of the extraction mechanism. Recognizing that simple measurement of absolute luminance is inherently unreliable post-print–scan due to global offsets introduced by scanner light, paper quality, and ink properties, the authors proposed using a composite decision statistic. This fuses multiple statistical features (such as mean luminance and sample variance) derived from an aggregate of text elements into a comprehensive index, allowing the embedded bit value to be determined via statistical hypothesis testing. This strategy, based on statistical moments rather than absolute values, significantly enhances robustness. Experimental validation across over ten thousand elements under various printer and scanner combinations confirmed the method’s strong cross-device robustness, proving TLM’s practical utility.

Building upon the success of TLM, the authors extended the technique to the color space, introducing Text Color Modulation (TCM) [30]. The core idea is to leverage multiple color channels (such as red, green, and blue in RGB, or cyan, magenta, yellow, and black in CMYK) to embed information. This permits multi-bit embedding per character (e.g., one bit encoded via the red channel intensity, another via the green). This multi-channel parallel encoding strategy dramatically enhances watermarking capacity. However, the application of TCM is subject to several constraints. Firstly, the scheme necessitates color printing, which significantly increases costs compared to grayscale for high-volume documentation. Secondly, achieving color consistency across different printers and scanners is challenging, often requiring complex color calibration. Thirdly, colored text may contravene formatting standards or compromise the solemnity of formal documents (e.g., contracts and certificates). Consequently, TCM is best suited for specific applications that are cost-insensitive, permit color aesthetics, and demand high capacity, such as high-end anti-counterfeiting or art provenance tracing.

Building upon the success of TLM, researchers introduced Text Halftoning Modulation (THM) [29]. Leveraging halftoning, a fundamental printing technique that simulates continuous tones through binary dot density, THM modulates a character’s perceived luminance by adjusting its halftoning pattern rather than its continuous grayscale value. This approach closely mirrors the actual printing process, as most printers fundamentally rely on halftoning for grayscale reproduction. Specifically, THM quantizes each character’s target luminance, using halftoning algorithms (such as error diffusion or ordered dithering) to generate a corresponding dot pattern whose density carries the watermark information. THM exhibits superior robustness over direct luminance modulation because halftoning patterns—the existence of a dot being more stable than a precise grayscale value—are better preserved during the print–scan process. Furthermore, halftoning aids imperceptibility, as the human visual system perceives luminance as a statistical average of the dot pattern. Comparative experiments confirm that THM surpasses TLM in both extraction accuracy and tolerance to print–scan distortion, representing a further maturation of luminance modulation techniques.

#### 3.1.3. Stroke-Based Modulation

Text characters, particularly those within ideographic systems such as Chinese and complex Latin scripts, exhibit rich stroke structures. Geometric attributes of these strokes, including width, orientation, length, and curvature, can be modulated within specific tolerances without compromising character legibility. Stroke-based watermarking methods [31,32] exploit this property by encoding the watermark payload directly into these geometric parameters. In contrast to pixel-level modifications, stroke-level alterations generally offer superior semantic consistency and enhanced robustness against geometric distortions.

Tan et al. [31] proposed a stroke direction modulation method tailored to the structural characteristics of Chinese characters. As these characters consist of fundamental strokes that can be rotated within specific ranges without compromising legibility, the authors utilized this feature for embedding. The process begins with extracting stroke centerlines via skeletonization and identifying modifiable segments. The orientation angles of these segments are quantized into discrete intervals, each corresponding to a bit value. Strokes are then rotated to align with the target interval, followed by morphological correction to ensure connectivity and naturalness. During extraction, the scanned document undergoes similar skeletonization and segmentation, and bit values are decoded by measuring stroke angles against the predefined intervals. Experiments demonstrate high extraction accuracy after print–scan and low-quality photocopying processes, alongside excellent imperceptibility. However, the method exhibits significant linguistic limitations due to its reliance on the complex structure of Chinese characters. For Latin scripts or other systems with simpler strokes, the scarcity of adjustable segments and restricted rotation ranges severely limit capacity and robustness, thereby hindering international application. Furthermore, the approach is critically dependent on stroke extraction performance, yet a fully mature and robust stroke extraction technique remains unavailable.

Compared to stroke orientation, stroke width represents a more universal feature, as characters across virtually all writing systems possess adjustable stroke thickness. Amano et al. [32] exploited this by proposing a stroke width modulation method. The core design employs a dual-partition strategy, where each character’s bounding box is uniformly divided into two complementary sets (Set 1 and Set 2). Two operations are defined: Fattening (F) and Thinning (T), which increase or decrease stroke width by adding or removing boundary pixels, respectively. During embedding, inverse operations are applied to the partitions: to embed a bit ‘1’, Set 1 is fattened while Set 2 is thinned; conversely, a bit ‘0’ is embedded. Accordingly, the extraction mechanism relies on differential statistics. Upon scanning, the total lengths of vertical black pixel runs within each partition are calculated as $S_{1}$ and $S_{2}$, yielding the difference $\Delta =S_{1}-S_{2}$. The value Δ is compared against a preset threshold *T*: if Δ>T, a bit ‘1’ is extracted; otherwise, a ‘0’ is determined. Experimental results demonstrate that this method achieves considerable robustness in print–scan scenarios and is applicable across various scripts. However, the method suffers from relatively low capacity, typically embedding only one bit per text line. Furthermore, reliable extraction requires characters to possess sufficient stroke density.

#### 3.1.4. Edge-Based Modulation

Character boundaries serve as the interface between the character and the background, encapsulating rich directional and morphological information. Edge-based watermarking schemes [33,55] exploit this by modulating geometric attributes or statistical features to embed data.

Specifically, Varna et al. [55] proposed a method based on left-edge position modulation. By defining two sets of feature pixels along the character’s left boundary, this approach alters the spatial distance between them by inserting or detecting pixels. The watermark payload is encoded based on the parity or threshold relationship of this distance; for instance, an odd distance represents a ‘1’, while an even distance denotes a ‘0’. The primary advantages of this scheme are its algorithmic simplicity and robustness against print–copy–scan operations. Since the method relies on relative distances rather than absolute coordinates, it exhibits inherent tolerance to translation, moderate scaling, and rotation. However, a significant drawback lies in its limited imperceptibility. The modification of edge pixels induces visible protrusions or indentations, which become notably conspicuous in high-resolution scans or displays. This visibility of artificial artifacts restricts the method’s applicability in scenarios requiring strict covertness.

Kim et al. [33] approached edge information from a statistical perspective by proposing a method based on Edge Direction Histogram (EDH) modulation. EDH serves as a statistical descriptor of the edge orientation distribution; it is constructed by detecting all edge points within an image, calculating their gradient directions, and aggregating the counts into quantized directional bins. The core strategy involves incrementally modifying pixels to precisely modulate the magnitude of specific bins, thereby enforcing the statistical constraints required for watermark encoding. Experimental results demonstrate the method’s strong generalization across diverse linguistic scripts without requiring language-specific tuning, representing a significant advantage over stroke-based techniques. However, the method suffers from relatively high computational complexity, as each embedding step necessitates iterative edge detection and histogram recalculation, which may become a bottleneck for large-scale document processing. Furthermore, the approach exhibits sensitivity to the selection of the edge detection algorithm (e.g., Canny, Sobel, and Prewitt); inconsistent implementations between embedding and extraction can yield divergent histograms, potentially leading to decoding errors.

Table 2 provides a comprehensive summary of other traditional watermarking approaches, namely color-based, stroke-based, and edge-based modulation methods. In addition to comparing their core embedding mechanisms, the table systematically evaluates their performance in terms of imperceptibility, generalizability, and capacity, while highlighting their robustness against common cross-media distortions, including print–scan, print–camera, and screen capture.

### 3.2. Deep Learning-Based Methods

With breakthroughs in deep learning for image representation, deformation modeling, and generation tasks, an increasing number of studies have introduced these techniques into the field of text image watermarking. These methods rely on end-to-end neural networks to complete the entire process of watermark embedding (Encoder), attack/degradation simulation (Distortion Module), and watermark extraction (Decoder). Consequently, they achieve high robustness under complex cross-media dissemination conditions (e.g., print-scan, print-camera, screenshooting).

StegaStamp [56] was one of the early works applying Deep Convolutional Neural Networks (CNNs) to image watermarking. The core idea is to construct an end-to-end trained autoencoder architecture; the encoder maps secret information to minute perturbations of image pixels, while the decoder recovers the original information from the watermarked image. By introducing the simulation of the print–camera process during the training phase, this method possesses a certain degree of robustness against physical media propagation. However, StegaStamp has two significant limitations: first, the watermark capacity is low, making it difficult to embed large amounts of information; second, the perturbations to the image are relatively obvious. Especially when the carrier is a text image, the visual artifacts after embedding are particularly prominent, which largely restricts its practicality in document watermarking scenarios.

Addressing the specificity of document images, Ge et al. proposed two targeted deep neural network watermarking schemes in 2023 [14,15]. The first scheme specifically addresses the increasingly prevalent threat of screen-shooting attacks. The key lies in the precise modeling of shooting noise and perspective transformations during the training phase. Through data augmentation and adversarial training strategies, the embedded watermark remains extractable within the screen–camera propagation link. The second scheme focuses on the local structural features of document images, utilizing deep neural networks to learn low-perturbation regions within the document and embedding binary watermark information into these locations that have minimal impact on visual quality. The loss function of this scheme carefully designs a trade-off mechanism between watermark recoverability and image structural fidelity. Nevertheless, experiments indicate that these two methods still produce relatively noticeable effects on the visual quality of the image after embedding, and traces of embedding can be perceived in certain cases. This suggests that achieving completely invisible deep learning watermarking on highly structured, low-redundancy carriers like document images remains extremely challenging.

A series of studies by Loc et al. provided new perspectives for the field of font watermarking [16,17]. Their first study was based on Generative Adversarial Networks (GANs), using deep generative models to embed secure information into document images. The core advantage is that the generative network can learn the natural texture distribution of the document, thereby generating controllable perturbations consistent with the original document style in local regions. This makes the embedded watermark more difficult to identify by the human eye or statistical detection methods. This strategy maintains the natural appearance of the document while ensuring information hiding. Their second work introduces a Fully Convolutional Network (FCN) architecture. Unlike networks with fixed input sizes, the fully convolutional nature of FCN allows it to directly process document images of arbitrary sizes without preprocessing steps like cropping or scaling. More importantly, through end-to-end learning, FCN can automatically discover the most suitable feature locations for watermark embedding, which often correspond to robust feature regions within the document. Experimental verification shows that the watermark embedded by this method maintains a high extraction accuracy after undergoing print–scan processes of varying quality. Both methods used by Loc et al. comprehensively achieved a relatively ideal balance between the two key metrics of robustness and invisibility, laying the foundation for the application of deep learning watermarking technology in actual document protection scenarios.

As summarized in Table 3, recent deep learning-based methods are compared across several dimensions, including their specific embedding mechanisms, imperceptibility, generalizability, and capacity. Furthermore, it explicitly highlights their robustness against common cross-media distortions, namely print–scan, print–camera, and screen capture.

## 4. Font-Based Text Watermarking

Font-based text watermarking embeds secret information by modulating the visual appearance of fonts, specifically by altering the geometric structure or parameters of characters. The fundamental principle involves generating a codebook composed of multiple character variants for each character; these variants are visually indistinguishable yet possess distinct subtle differences, with each variant mapping to a specific binary symbol or bit sequence. The embedding process entails substituting characters in the original text with their corresponding variants according to the payload. Compared to image-based pixel-level modifications, font-based approaches generally offer superior embedding capacity while preserving semantic integrity, as each character can independently encode one or more bits. This capacity advantage renders the technique uniquely valuable for applications requiring the embedding of lengthy identifiers or complex metadata, thereby establishing it as a prominent research focus in recent years. Based on the mechanism of variant generation, existing methods can be systematically categorized into two classes: hand-crafted approaches and automated generation approaches, as shown in Figure 2. The former relies on manual intervention to construct watermarked fonts, such as manually modifying character structures or selecting visually similar variants. Conversely, the latter leverages machine learning, optimization algorithms, or generative models to automatically synthesize watermarked variants from source fonts. The following sections provide a detailed review of representative works within these two categories.

### 4.1. Hand-Crafted Methods

Hand-crafted methods [34,35,36] are characterized by the integration of human visual perception and design expertise into the generation and selection of glyph variants. This approach prioritizes ensuring that the resulting variants remain visually congruent with the original characters, thereby preserving the imperceptibility of the watermark.

FontCode [34] was the first work to systematically employ the concept of a font manifold for watermark embedding. Building on the theoretical framework of Campbell and Kautz [57], which suggests that fonts exhibiting similar visual characteristics occupy a low-dimensional manifold within a high-dimensional feature space, FontCode leverages this structure to generate controlled glyph variations. By introducing subtle perturbations to original glyphs in the manifold space, the method produces variants that are visually indistinguishable from the source while remaining separable in the encoding space. This manifold-driven approach preserves the stylistic coherence of the original font and provides sufficient distinctiveness for reliable extraction. The workflow consists of several steps. The manifold representation of the target font is first learned. Perturbed versions are then produced within a local neighborhood of the manifold, with each version corresponding to a specific bit value. Human inspection is subsequently required to select visually acceptable variants for constructing the final codebook. During embedding, characters in the text are replaced with the corresponding variants according to the watermark bit sequence. Extraction relies on a trained classifier that identifies the variant category of each character and recovers the embedded payload. Although conceptually innovative, FontCode exhibits several notable limitations. (1) Its robustness is limited, especially when the watermarked text undergoes physical processes such as printing and scanning, where ink diffusion and scanning noise may obscure the subtle perturbations and cause extraction failure. (2) Its extensibility across fonts and languages is also restricted. For scripts with complex stroke structures, such as Chinese or Japanese, learning a stable manifold and generating reliable perturbations becomes significantly more challenging. (3) The construction of the codebook requires manual screening of glyph variants, which is time-consuming and subjective, making large-scale deployment difficult. (4) In addition, the method requires training a separate classifier for each character, which increases the overall system complexity and renders the approach impractical for large character sets such as those containing thousands of Chinese characters.

Qi et al. proposed a simplified template matching-based scheme. The method still follows a hand-crafted design philosophy but streamlines the extraction process. Instead of relying on a complex classifier, it identifies the used glyph variant through template matching. For each character and each variant, a reference template is created in advance. During extraction, the scanned character is compared with all templates, and the bit value corresponding to the template with the highest similarity is returned as the decoding result. The limitations are clear. The cost of manually constructing a font library is high, especially for languages with large character sets. The watermark capacity is limited because the number of manually designed variants is usually small. The robustness of template matching is also insufficient when facing geometric transformations such as rotation, scaling, or perspective distortion, which can be problematic in practical use. It is important to note that both approaches rely heavily on OCR to identify the character before performing variant classification or template matching. This strong dependence on OCR introduces instability in extraction accuracy. When document quality is low, when rare or unconventional fonts are used, or when multiple languages appear in the same document, OCR errors propagate directly to the watermark extraction stage and cause cascading failures. This issue has received increasing attention in subsequent research.

Considering the unique characteristics of Chinese characters, including complex stroke structures, a large character inventory, and diverse visual forms, Yao et al. [36] proposed a font watermarking method specifically designed for Chinese script. The method adopts a perturbation transfer strategy. It begins by manually creating a base perturbed font through carefully designed microscopic modifications applied to a reference font. A font generation model, such as a deep learning-based style transfer network, is then used to transfer this perturbation pattern to other fonts. This process enables large-scale generation of watermarked variants and results in a Chinese oriented font watermark library. The method offers several advantages. The perturbation transfer mechanism allows the watermarking capability to be extended to multiple Chinese fonts with relatively high efficiency, without the need to design perturbations for each font individually. The approach is also optimized for the complexity of the Chinese character set by considering stroke connectivity and structural balance. Experiments show that the generated variations introduce minimal visual changes and achieve good imperceptibility. Moreover, the inherent visual complexity of Chinese characters provides a relatively large embedding capacity. Despite these strengths, the method has several limitations. The construction of the font library remains complex and requires careful design of the base perturbation pattern as well as tuning of the font generation model. In addition, the overall performance of the system is highly dependent on the quality of the perturbation design, which still relies on the designer’s experience and aesthetic judgment.

### 4.2. Automatically Generated Methods

In contrast to hand-crafted approaches, automated generation methods [37,38,39,40,41,42,43] seek to algorithmically identify optimal glyph perturbation strategies, thereby minimizing manual intervention while enhancing scalability and systematicity. Typically leveraging machine learning, optimization algorithms, or generative models, these techniques enable the end-to-end learning of mappings from source fonts to their watermarked counterparts.

Wang et al. [37] proposed the “fusion fonts” method, a scheme specifically engineered to withstand print–scan attacks. The core methodology leverages style transfer and Generative Adversarial Networks (GANs) to integrate visual features from multiple source fonts, generating novel typefaces with inherent encoding capabilities. The workflow comprises selecting source fonts to extract stylistic features, employing a GAN framework to synthesize new glyphs through learned feature combinations, and substituting the original document fonts to implement watermark embedding via a predefined coding mechanism. This approach offers distinct advantages, notably a higher embedding capacity per character compared to traditional pixel-flipping techniques and substantial resilience against print–scan distortions due to the inclusion of degradation characteristics during generation. However, several limitations persist. First, font generation and substitution may impair typesetting compatibility; generating complete libraries for large character sets, such as Chinese, demands extensive computational resources and risks rendering anomalies in certain layout engines. Second, while optimized for print–scan channels, the method’s robustness against complex cross-media transmission scenarios, including screen photography, JPEG compression, and social media re-encoding, lacks comprehensive evaluation.

Addressing the vector characteristics of Chinese fonts, Qi et al. [38] proposed a method to generate watermarked font libraries by automatically modifying vector contour curves. The workflow is as follows: the original vector contour data of the character glyphs is obtained; second, the contours are refined to extract skeleton curves, which provide an abstract representation of the glyphs’ topological structure; third, key control points on the skeleton are identified and filtered according to specific rules, representing locations that can be safely altered without compromising glyph recognizability; finally, the vector contours are automatically modified around these selected points, and the resulting glyphs are stored in a new font library. This vector-based approach offers several advantages. The resolution-independent nature of vector fonts ensures that the modified glyphs can be rendered at any size without quality loss. Manipulating vector control points enables more precise and controllable perturbations than raster-based modifications. The automated selection and modification of key points reduces manual intervention, enhancing scalability. However, the method has limitations. The design of the modification rules relies on domain knowledge and experience, and the parameters may require adjustment for different font styles. Moreover, for highly complex or unconventional glyphs, automatic key-point detection may fail, resulting in suboptimal variant quality.

In recent years, Yang et al. proposed AutoStegaFont [39] and its improved version AutoStegaFont+ [40], representing the latest advances in automated font-based watermarking. Both methods adopt a dual-modality framework to synthesize vector fonts that encode information through end-to-end learning. The framework is built around a two-stage training strategy. In the first stage, the encoder and decoder networks are jointly trained, with the encoder learning to embed watermark information into the latent representation of glyphs and the decoder learning to extract the information from the watermarked glyphs. This stage operates on glyph images. In the second stage, glyph images are converted into vector fonts based on the embedded bit information. The main advantages of the AutoStegaFont series are as follows. First, it achieves high robustness across multiple font types, including English fonts and Chinese Songti, with experimental results showing significantly higher extraction accuracy under print-and-scan scenarios compared with traditional methods. Second, the dual-modality framework balances the expressive power of deep learning with the editability of vector fonts. Third, the end-to-end optimization reduces manual intervention and enhances automation. Nevertheless, the method has notable limitations. The training process is highly complex, requiring large amounts of glyph data and substantial computational resources. Furthermore, a complete two-stage training procedure must be repeated for each new font, limiting the method’s practicality in scenarios that demand rapid adaptation across multiple fonts.

Sun et al. [41] proposed a fully automated font watermarking method that does not require any labeled data. Specifically, the method first learns a manifold representation of the target font in the style space and then introduces directional perturbations within the local neighborhood of the manifold, allowing the modified glyphs to encode watermark information. However, the method exhibits a significant limitation. It lacks a systematic quantitative evaluation of visual quality. The watermark residual maps presented in the paper reveal substantial visual distortions in the glyphs, with perturbations in certain strokes clearly exceeding human perceptual thresholds. This indicates that the method may underperform in terms of invisibility compared with other carefully designed approaches, representing a serious drawback in applications requiring high concealment. In addition, the paper provides limited comparative experiments with existing methods, making it difficult to objectively assess its practical performance.

In the same year, He et al. [42] proposed a font variant generation method based on centroid-adaptive offsets. The core idea is to create glyph variants by slightly shifting the centroid of each glyph, with different offset directions or magnitudes representing different watermark bits. In practice, the method uses an optimization algorithm to determine which pixels or vector control points should be adjusted, ensuring that the glyph centroid moves precisely to the target position while minimizing visual distortion. During extraction, the watermark is decoded by measuring the centroid positions and comparing them with reference positions. Despite its novelty, the method has significant limitations. Its evaluation is restricted to a single transmission channel, namely digital screenshots, and lacks systematic testing across physical media such as print-and-scan or print-and-photograph scenarios. Therefore, its robustness in practical document protection applications remains uncertain.

FontGuard [43] represents the latest advancement in font watermarking. Unlike previous methods that primarily focus on pixel-level modifications, FontGuard introduced a font model and a language-guided contrastive learning mechanism. Its key innovations are as follows. First, watermark embedding is performed by modifying the hidden style feature space rather than directly manipulating pixels or vector control points. Alterations in this high-level semantic space are more robust, as style features exhibit invariance to low-level noise and distortions. Second, FontGuard exploits the richness of the font manifold to generate numerous visually similar glyph variants, achieving high embedding capacity. Third, during decoding, a contrastive learning framework is employed to learn invariant representations of glyphs under various distortion conditions, significantly enhancing robustness against real-world transmission distortions. Experiments demonstrate improvements in robustness under synthetic, cross-media, and online social network transmission scenarios, with a 52.7% improvement in visual quality measured by LPIPS. Nevertheless, FontGuard has a potential limitation. The method lacks systematic evaluation on print-and-scan, the most common cross-physical media scenario. This omission is noteworthy, as distortions introduced by printing and scanning may effectively disrupt or remove the hidden style features, potentially causing watermark extraction failures. Consequently, the practical performance of FontGuard in traditional document protection applications remains to be validated.

Table 4 presents a detailed summary of font-based watermarking techniques. By comparing their core embedding mechanisms, the table benchmarks the effectiveness of modifying font representations. It assesses each method’s imperceptibility, generalizability, and capacity, alongside its specific cross-media robustness to print–scan, print–camera, and screen capture scenarios.

## 5. Format-Based Text Watermarking

Format-based text watermarking [44,45,46,47,48,49,50,51,52] embeds secret information by modifying document layout parameters. The underlying principle of format-based watermarking is to exploit the redundant degrees of freedom in document layout, which are parameters that can vary within a limited range without affecting readability or visual aesthetics, to encode information. From a technical perspective, format-based text watermarking primarily consists of two core methods: line spacing modulation and word spacing modulation. Line spacing modulation encodes information by adjusting the vertical distance between text lines, while word spacing modulation embeds information by altering the horizontal distance between words.

Research on format-based text watermarking dates back to 1995, when Brassil et al. [44] first systematically proposed the idea of encoding information in documents by shifting the positions of text lines or words. This work defined two fundamental encoding strategies: line-shift coding and word-shift coding. The principle of line-shift coding is to select certain text lines as embedding lines and apply small vertical displacements relative to their reference positions according to the watermark bit values; for example, an upward shift representing bit ‘0’ and a downward shift representing bit ‘1’, or vice versa. A notable advantage of line-shift coding is its support for blind detection. During watermark extraction, the original document is not required; the watermark can be decoded by analyzing the relative spacing between lines in the scanned document. This is possible because line spacing is generally uniform, and anomalous variations can be detected through statistical analysis. This property makes line-shift coding practical, as storing or transmitting the original document is often unfeasible. Word-shift coding is conceptually similar but operates at the word level. It encodes watermark bits by slightly adjusting the horizontal positions of specific words relative to their neighbors. However, word-shift coding faces a fundamental challenge: in typical document layouts, word spacing is inherently non-uniform. This non-uniformity arises from various layout mechanisms, such as justification, which automatically adjusts word spacing to fill line width, and proportional fonts, where character widths vary. Unlike line-shift coding, word-shift coding generally requires either the original text or a statistical model of word spacing as a reference to reliably distinguish natural spacing variations from watermark-induced changes. This reliance on reference information significantly reduces the practicality of word-shift coding.

Maxemchuk, Nicholas F. et al. [45,46] conducted a systematic comparison of these two methods and concluded that line-shift coding is more resistant to distortions during transmission across different media. Although word-shift coding is less perceptible, it relies on precise word-level positioning and is therefore more vulnerable. In terms of security, line-shift coding has a notable weakness because its modifications are relatively easy to detect and remove. The vertical shifts in lines often exhibit visually regular patterns, such as odd lines moving upward and even lines moving downward, which can be identified by a deliberate attacker through statistical analysis. The watermark can then be removed through reformatting. Word-shift coding offers an advantage in this regard, as the subtle variations in word spacing are more concealed and thus more difficult to detect and attack.

The concepts of line-shift coding and word-shift coding have inspired further research aimed at optimizing these algorithms. Huang et al. [47] introduced a signal processing perspective and proposed an innovative approach that models variations in word spacing as continuous sinusoidal signals rather than discrete jumps. The advantages of this method are twofold. Sinusoidal spacing variations are less perceptible to the human eye, enhancing invisibility, and watermark information can be encoded in the phase, amplitude, and frequency of the sinusoid, making it statistically detectable. Although this method supports blind detection, its embedding capacity remains limited.

Alattar et al. [48] introduced spread spectrum techniques and BCH error-correcting codes (Bose–Chaudhuri–Hocquenghem codes) to enhance the robustness of line-shift and word-shift coding. However, the increase in robustness comes at the cost of reduced embedding capacity, resulting in performance that is not ideal and, in some cases, even lower than that of the method proposed by Huang et al. [47].

Zou and Shi [49] proposed the Inter-word Space Modulation (ISM) algorithm, a simple yet effective word-spacing adjustment scheme. The core idea of ISM is to divide the spacing between all adjacent word pairs in a document into two non-overlapping categories: narrow spacing and wide spacing. During watermark embedding, the spacing of specific word pairs is slightly adjusted according to the bit to be embedded, so that it falls into the corresponding category. For example, to embed bit ‘0’, the spacing is adjusted to the narrow range; to embed bit ‘1’, it is adjusted to the wide range. A safety margin is maintained between the two categories to ensure correct classification even if spacing changes slightly during the print-and-scan process. During extraction, the spacing between words in the scanned document is measured to determine the category, thereby decoding the embedded bit. The method exhibits high robustness to print-and-scan operations, as it relies on relative spacing rather than absolute values, and the binary classification boundaries can be dynamically adjusted using adaptive thresholding to accommodate different scanning qualities. However, a notable limitation of ISM is its very low embedding capacity. Typically, only one bit can be embedded per line of text, because the method employs line-level coding in which the spacing pattern of an entire line collectively represents a single bit to enhance robustness. This low capacity is insufficient for applications requiring the embedding of long identifiers or complex metadata.

To address the limited capacity issue, Culnane et al. [50] proposed an improvement based on the work of Zou and Shi. Their main innovation is to treat the entire document as a single continuous line rather than processing each line independently, thereby utilizing all available spacing and eliminating the previous use of “spare sets,” which significantly increases embedding capacity. The study introduced a frequency distribution-based directionally weighted nearest-neighbor thresholding method to accurately distinguish between inter-letter and inter-word spacing. Additionally, frequency shaping was employed to adjust inter-letter spacing to one pixel after embedding, with the adjustment uniformly distributed to maintain consistent line length. Experiments conducted at 150 dpi and 300 dpi using nine fonts and three watermark patterns demonstrated an average capacity increase of approximately 20 percent. Xia et al. [51] proposed two robust watermarking methods for Chinese text images. The first method embeds the watermark by modulating inter-character spacing, grouping words, and creating detectable differences between groups. The second method encodes information based on relative character height, shifting characters vertically to deviate from a reference line. Both methods employed a specialized Chinese character segmentation algorithm that combines horizontal and vertical projection with connected component analysis and includes optimizations for square-shaped characters and left–right structural features. Preprocessing involved iterative binarization and skew correction based on interline white space. Experiments on 60-page documents of various fonts and sizes printed and scanned at 600 dpi showed high extraction accuracy, an embedding capacity of approximately 1.5 bits per line, and strong robustness against distortions introduced by the print-and-scan process.

Table 5 presents a detailed summary of format-based watermarking techniques. It outlines how different approaches manipulate document layouts as their core embedding mechanisms, and assesses their overall imperceptibility, generalizability, and capacity. Additionally, the table highlights their resilience across various cross-media scenarios, including print–scan, print–camera, and screen capture.

## 6. Open Challenges and Future Directions

To provide a more coherent overview, Table 6 summarizes the strengths and weaknesses of the major technical routes discussed in this survey, along with the data types they primarily target. Traditional image-based methods and deep learning-based approaches primarily operate on the pixel level, making them highly effective for raw document images where layout or font information is unavailable. However, they often struggle with the low-pixel redundancy of text, where deep learning models may introduce noticeable artifacts, and traditional methods face a sharp trade-off between robustness and invisibility. In contrast, font-based and format-based techniques shift the embedding locus to structural and layout parameters. Font-based methods like AutoStegaFont and FontGuard offer significantly higher embedding capacity by exploiting glyph manifolds, but they struggle to achieve universality across different fonts and languages. Meanwhile, format-based methods prioritize content integrity and are nearly invisible, yet they remain highly vulnerable to re-typesetting attacks and offer the lowest data payload.

In the following subsections, we will delve into the specific challenges and future research directions for each category of text watermarking in detail.

### 6.1. Image-Based Text Watermarking

#### 6.1.1. Open Challenges

Traditional feature modulation methods implement watermark embedding through carefully designed microscopic adjustments to the physical or geometric attributes of text images. These methods share several distinct advantages: high interpretability, where operational objectives and effects are transparent; low computational complexity, typically involving fundamental image processing operations; and established security analysis frameworks grounded in information theory and cryptography. However, they also face common challenges. First is the inherent trade-off between robustness and imperceptibility. Substantial feature modulation is required to withstand physical channel attacks like print–scan, yet this inevitably exacerbates visual artifacts. Conversely, prioritizing perfect imperceptibility often compromises robustness. Optimizing this equilibrium remains an open research question. Second is the conflict between capacity and visual quality. High-capacity embedding necessitates modifying a greater number of pixels or character features, which cumulatively degrades visual fidelity. Developing efficient coding schemes to maximize payload within a constrained modification budget is crucial for enhancing utility. Third is the dependency on specific languages or fonts. Many techniques, particularly stroke-based approaches, rely heavily on specific scripts or font styles, hindering their generalization to other writing systems. Consequently, developing universal, language-agnostic approaches is a prerequisite for international application.

#### 6.1.2. Future Directions

Looking forward, a promising direction for image-based text watermarking lies in the deeper integration of traditional feature modulation paradigms with learning-based methods [58]. In such hybrid frameworks, deep learning models can be leveraged to automatically identify content-aware and robust modulation regions, while traditional techniques perform precise and interpretable modifications. This combination offers a principled way to balance robustness, imperceptibility, and explainability, addressing several long-standing challenges of purely hand-crafted approaches.

Building upon this paradigm, recent advances in generative modeling, particularly diffusion-based models, further expand the design space of learning-driven watermarking. Owing to their strong generative capacity and fine-grained control over image synthesis, diffusion models have the potential to enable more flexible and content-adaptive watermark embedding. In particular, their progressive denoising process may facilitate accurate modulation of local visual details, offering opportunities to better align watermark perturbations with document texture distributions and perceptual characteristics.

Nevertheless, fully realizing these potential benefits remains non-trivial and requires further investigation. Diffusion models rely on iterative stochastic sampling, which poses challenges in ensuring deterministic and repeatable watermark signals—an essential requirement for reliable extraction under cross-media transmission such as print–scan or print–camera processes. In addition, their high computational cost and latency constrain their applicability in large-scale document processing and real-time watermarking scenarios. More importantly, text images exhibit highly discrete and low-redundancy structures, where even minimal perturbations may lead to visible artifacts or character deformation. How to consistently enforce strict imperceptibility constraints throughout the diffusion trajectory, while preserving watermark robustness and stability, therefore remains an open research problem.

Beyond architectural choices, the deeper integration of perceptual models, such as Human Visual System (HVS) or OCR response models, into the watermarking design pipeline holds significant potential for further enhancing imperceptibility. Finally, given the diverse attack channels encountered in real-world applications, the development of adaptive strategies that dynamically adjust embedding parameters based on document content and anticipated usage scenarios will be instrumental in improving practical viability.

### 6.2. Font-Based Text Watermarking

#### 6.2.1. Open Challenges

Font-based text watermarking methods embed information by modifying glyph appearances, offering semantic-level advantages and greater capacity potential compared with pixel-based image modifications. The field has evolved from manual design to automated generation, marking a paradigm shift from reliance on human expertise to data-driven learning. Manual design methods provide high controllability and interpretability but suffer from limited scalability and automation. Automated generation approaches offer greater potential for large-scale applications, yet they often face challenges such as high training complexity and difficulty in adapting to new fonts. The core challenges in the field include the following. First, universality: designing methods that can rapidly adapt to different languages and font styles without requiring complex training or adjustments for each font is difficult. Second, comprehensive robustness: existing methods are typically optimized for specific transmission channels, such as purely digital media or certain physical media, lacking robustness across diverse attack scenarios. Third, the trade-off between invisibility and capacity: maximizing embedding capacity while preserving glyph visual quality remains an open problem.

#### 6.2.2. Future Directions

Looking forward, several promising research directions can be identified. One is leveraging advances in large-scale font generation models, such as diffusion models or transformers, to produce higher-quality and more controllable glyph variants. Another is incorporating advanced perceptual models, such as quality assessment networks based on human visual attention, to guide watermark embedding and ensure invisibility. A third direction involves designing unified training frameworks for multi-distortion robustness, enabling a single model to withstand both digital and physical media distortions. Finally, exploring few-shot or zero-shot font watermarking schemes could allow rapid adaptation to new fonts with minimal or no training. Progress in these areas is expected to propel font-based text watermarking toward practical, real-world applications.

### 6.3. Format-Based Text Watermarking

#### 6.3.1. Open Challenges

Format-based text watermarking methods embed information by modifying document layout parameters, with the key advantage of preserving textual content integrity, which is indispensable in many content-sensitive applications. The main challenges in this field include the following. First, there is an inherent trade-off between capacity and robustness, as almost all techniques that improve robustness, such as spread spectrum, error-correcting codes, or multi-sample averaging, incur a reduction in embedding capacity. Second, these methods are highly dependent on the underlying typesetting system and document format. Format-based watermarking typically assumes a consistent layout, but in practice, different word processing software, PDF rendering engines, or printer drivers can produce varying layout results, leading to inconsistencies in embedding and extraction. Third, many methods require high-precision image processing, relying on pixel-level measurements and positioning, which may not be reliable under low-quality scanning or photography conditions.

#### 6.3.2. Future Directions

Looking forward, several promising directions can be explored. One is the use of deep learning-based adaptive modulation strategies, where neural networks learn optimal spacing adjustment patterns for a given document and distortion model, enabling a dynamic balance between capacity and robustness. Another is multimodal fusion, combining format-based watermarking with image- or font-based watermarking to exploit the complementary strengths of different carriers and enhance overall performance. A third direction is the development of format-independent watermarking schemes, ensuring that watermarks remain intact even when documents undergo format conversions, such as PDF to Word or printing followed by digitization. Advances in these areas are expected to expand the practical applicability of format-based text watermarking in copyright protection, document tracing, and authentication.

### 6.4. Attack Models and Security Considerations

As a security-oriented technique, text watermarking must be evaluated not only in terms of robustness and imperceptibility, but also under explicit adversarial threat models. Different watermarking paradigms provide fundamentally different security properties, and their reliability depends critically on the types of attacks they are designed to resist. In this section, we discuss representative attack models relevant to semantic-preserving text watermarking and analyze their implications across different method categories.

#### 6.4.1. Removal Attacks

Removal attacks aim to eliminate or weaken embedded watermarks without necessarily preserving the original content or layout. In semantic-preserving text watermarking, common removal operations include document reformatting, reprinting, resampling, binarization, smoothing, and geometric normalization. Format-based watermarking methods are particularly vulnerable to such attacks, as re-typesetting or layout reflow can easily destroy spacing-based signals. Image-based approaches, especially those designed for print–scan robustness, typically exhibit stronger resistance to removal attacks due to their reliance on relative structural or statistical invariants. Font-based watermarking methods occupy an intermediate position: while font substitution or glyph normalization may remove watermarks, attacks that preserve font identity often retain the embedded signals.

#### 6.4.2. Forgery Attacks

Forgery attacks attempt to create counterfeit documents that either replicate a valid watermark or falsely attribute ownership by transplanting watermark signals from one document to another. Such attacks pose a significant challenge for watermarking schemes that lack content binding or contextual dependency. Image-based watermarking methods that encode information relative to local structure or spatial context generally provide stronger resistance to naive transplantation, whereas format-based methods may be more susceptible due to their global and repetitive nature. Font-based watermarking approaches can mitigate forgery risks when watermark encoding is tightly coupled to glyph-specific or font-specific characteristics, although this often comes at the cost of reduced generalizability.

## 7. Conclusions

In this paper, we have presented a comprehensive survey of semantic-preserving text watermarking technologies, addressing a critical gap in the literature regarding methods that ensure both content integrity and visual consistency. While the recent surge in Large Language Models has directed significant attention toward linguistic steganography, our analysis highlights the indispensable role of image-based, font-based, and format-based approaches. These semantic-preserving methods provide crucial security for scenarios where semantic modification is unacceptable, such as in legal documents and official archives, successfully tackling the low-redundancy challenges of textual images. Through a unified classification framework, we have systematically evaluated existing techniques across key performance dimensions, including embedding mechanisms, robustness against physical and digital distortions, embedding capacity, and imperceptibility. By providing a deep understanding of these diverse embedding mechanisms, we hope to guide the community toward developing more comprehensive and versatile solutions, specifically in four directions: Universal Robustness: Moving from single-channel optimization (e.g., only print–scan) to unified frameworks capable of withstanding cross-media distortions (print–camera, screen capture, and re-compression) simultaneously. Multimodal Fusion: Developing hybrid schemes that combine font-level perturbations with spacing modulation to maximize embedding capacity without sacrificing visual quality. AI-Driven Interpretability: Integrating deep learning’s adaptive capabilities with traditional feature modulation to ensure that watermarking remains both robust and explainable for legal and forensic use. Zero-Shot Adaptation: Leveraging large-scale generative models (e.g., diffusion or transformers) to achieve font-agnostic watermarking that requires no prior training on new typefaces.

## Figures and Tables

**Table 1:** Summary of pixel-based modulation methods (PS—print–scan; PC—print–camera; SC—screen capture).

Article	Embedding Mechanism	Robustness			Imperceptibility	Generalizability	Capacity
		PS	PC	SC			
Wu et al. [18]	Flipping pixels based on smoothness and connectivity scores	✓	×	×	High	High	Medium
Qi et al. [19]	Modulating the character Black Pixel Count (BPC) ratio	✓	×	×	High	High	Low
Hou et al. [20]	Modulating local density in multi-resolution thumbnails	✓	×	×	High	High	Low
Tan et al. [21]	Flipping boundary pixels determined by Fourier descriptors	✓	×	×	Medium	High	High
Yang et al. [22]	Centroid shift encoding via stroke boundary modulation	✓	✓	✓	High	High	Medium
Li et al. [53]	Flipping specific boundary pixels	✓	×	×	Medium	High	Low
Meng et al. [1]	Modulating counts of continuous black pixel segments (COREs)	✓	✓	✓	High	High	High
He et al. [23]	Encoding in sign patterns of high-frequency DCT coefficients	✓	×	×	High	High	Low
Yang and Kot [25]	Flipping pixels based on topological invariants conservation	×	×	×	High	High	High
Yang and Kot [27]	Interleaved Morphological Binary Wavelet Transform (IMBWT) encoding	×	×	×	High	High	High
Wang et al. [26]	7-ary encoding using specific 2×2 block patterns	×	×	×	High	High	High

**Table 2:** Summary of color-based, stroke-based, and edge-based modulation methods. (PS—print–scan; PC—print–camera; SC—screen capture).

Article	Embedding Mechanism	Robustness			Imperceptibility	Generalizability	Capacity
		PS	PC	SC			
Borges et al. [28]	Modulating character luminance gain within defined ranges	✓	×	×	High	High	Low
Borges et al. [30]	Multiplexing information into multiple color channels (RGB/CMYK)	✓	×	×	High	High	High
Villan et al. [29]	Modulating halftoning dot density patterns	✓	×	×	High	High	Low
Tan et al. [31]	Quantizing orientation angles of stroke segments	✓	×	×	Medium	Low	High
Amano et al. [32]	Modulating stroke width via fattening and thinning operations	✓	×	×	Medium	High	Low
Varna et al. [55]	Modulating spatial distance between left-boundary feature pixels	✓	×	×	Low	High	High
Kim et al. [33]	Modulating the magnitude of Edge Direction Histogram (EDH) bins	✓	×	×	High	High	Low

**Table 3:** Summary of deep learning-based methods (PS—print–scan; PC—print–camera; SC—screen capture).

Article	Embedding Mechanism	Robustness			Imperceptibility	Generalizability	Capacity
		PS	PC	SC			
Tancik et al. [56]	End-to-end autoencoder mapping information to minute pixel perturbations	✓	✓	×	Low	High	Low
Ge et al. [15]	Adversarial training modeling shooting noise and perspective transformations	✓	✓	×	Low	High	Medium
Ge et al. [14]	Embedding in learned low-perturbation regions to preserve local structure	×	×	×	Low	High	Medium
Loc et al. [17]	Generating controllable perturbations consistent with document texture via GANs	✓	×	×	High	High	Medium
Loc et al. [16]	Fully Convolutional Network identifying robust feature locations for embedding	✓	✓	×	High	High	High

**Table 4:** Summary of font-based watermarking methods (PS—print–scan; PC—print–camera; SC—screen capture).

Article	Embedding Mechanism	Robustness			Imperceptibility	Generalizability	Capacity
		PS	PC	SC			
Xiao et al. [34]	Generating controlled glyph variations within a learned font manifold	✓	×	×	Medium	Low	High
Qi et al. [35]	Template matching of manually designed glyph variants	✓	×	✓	Medium	Low	High
Yao et al. [36]	Transferring perturbation patterns to target fonts via style transfer networks	✓	✓	×	Medium	High	High
Wang et al. [37]	Synthesizing fusion fonts by integrating visual features from multiple sources	✓	×	×	Medium	Low	High
Qi et al. [38]	Automatically modifying vector contour curves around key control points	✓	×	×	High	Medium	High
Yang et al. [39]	Dual-modality framework synthesizing vector fonts via end-to-end learning	✓	✓	✓	High	Low	High
Sun et al. [41]	Introducing directional perturbations in the style manifold space	✓	×	✓	Medium	High	High
He et al. [42]	Shifting glyph centroids using centroid-adaptive offsets	×	×	✓	Medium	Low	High
Wong et al. [43]	Modifying hidden style feature space via language-guided contrastive learning	×	✓	✓	Medium	High	High

**Table 5:** Summary of format-based watermarking methods (PS—print–scan; PC—print–camera; SC—screen capture).

Article	Embedding Mechanism	Robustness			Imperceptibility	Generalizability	Capacity
		PS	PC	SC			
Brassil et al. [44]	Shifting positions of text lines or words	✓	×	×	High	High	Low
Huang et al. [47]	Modulating word spacing as continuous sinusoidal signals	✓	×	×	High	High	Low
Alattar et al. [48]	Enhancing spacing modulation with spread spectrum and error correction	✓	×	×	High	High	Low
Zou and Shi [49]	Classifying inter-word spacing into narrow and wide categories	✓	×	×	High	High	Low
Culnane et al. [50]	Treating document as a continuous line to utilize all spacing	✓	×	×	High	High	Low
Xia et al. [51]	Modulating inter-character spacing or height for Chinese characters	✓	×	×	High	Low	Low

**Table 6:** Comprehensive comparison of semantic-preserving text watermarking methodologies.

Methodology	Data Type	Strengths	Limitations
Image-based	Binary/Grayscale	Low computational cost.	Robustness–invisibility trade-off;
(Traditional)	Text Images		
Image-based	Document	End-to-end robustness;	High complexity; Visual artifacts
(Deep learning)	Images	Handles complex documents.	on structured text carriers.
Font-based	Digital Fonts/	High embedding capacity;	High OCR dependence; Difficult
	Vector Graphics	Strong robustness.	cross-language generalization.
Format-based	Digital Docs/	Zero content modification;	Extremely low capacity;
	Formatted PDFs	High invisibility.	Easy removal.

## Data Availability

Data are contained within the article.

## References

[1] Meng J., Li Y., Lu Z., He Z., Luo H., Zhang T. (2025). CoreMark: Toward Robust and Universal Text Watermarking Technique. arXiv.

[2] Hu R., Zhang J., Zhao S., Lukas N., Li J., Guo Q., Qiu H., Zhang T. Mask Image Watermarking. Proceedings of the Thirty-Ninth Annual Conference on Neural Information Processing Systems.

[3] Sander T., Fernandez P., Durmus A., Furon T., Douze M. Watermark Anything with Localized Messages. Proceedings of the International Conference on Learning Representations (ICLR).

[4] Liu A., Pan L., Lu Y., Li J., Hu X., Zhang X., Wen L., King I., Xiong H., Yu P. (2024). A survey of text watermarking in the era of large language models. ACM Comput. Surv..

[5] Kamaruddin N.S., Kamsin A., Por L.Y., Rahman H. (2018). A review of text watermarking: Theory, methods, and applications. IEEE Access.

[6] Yang Z., Zhao G., Wu H. (2025). Watermarking for large language models: A survey. Mathematics.

[7] Liang Y., Xiao J., Gan W., Yu P.S. (2024). Watermarking techniques for large language models: A survey. arXiv.

[8] Xu Z., Yue X., Wang Z., Liu Q., Zhao X., Zhang J., Zeng W., Xing W., Kong D., Lin C. (2025). Copyright Protection for Large Language Models: A Survey of Methods, Challenges, and Trends. arXiv.

[9] Kirchenbauer J., Geiping J., Wen Y., Katz J., Miers I., Goldstein T. A watermark for large language models. Proceedings of the 40th International Conference on Machine Learning.

[10] Dathathri S., See A., Ghaisas S., Huang P.S., McAdam R., Welbl J., Bachani V., Kaskasoli A., Stanforth R., Matejovicova T. (2024). Scalable watermarking for identifying large language model outputs. Nature.

[11] Yang X., Zhang J., Chen K., Zhang W., Ma Z., Wang F., Yu N. (2022). Tracing text provenance via context-aware lexical substitution. Proc. AAAI Conf. Artif. Intell..

[12] He X., Xu Q., Zeng Y., Lyu L., Wu F., Li J., Jia R. (2022). CATER: Intellectual property protection on text generation APIs via conditional watermarks. Proceedings of the 36th International Conference on Neural Information Processing Systems.

[13] Xie G., Liu Y., Xin G., Yang P. (2019). Review on text watermarking resistant to print-scan, screen-shooting. Artificial Intelligence and Security.

[14] Ge S., Xia Z., Fei J., Tong Y., Weng J., Li M. (2023). A robust document image watermarking scheme using deep neural network. Multimed. Tools Appl..

[15] Ge S., Fei J., Xia Z., Tong Y., Weng J., Liu J. (2023). A screen-shooting resilient document image watermarking scheme using deep neural network. IET Image Process..

[16] Loc C.V., Burie J.C., Ogier J.M. (2018). Document images watermarking for security issue using fully convolutional networks. Proceedings of the 2018 24th International Conference on Pattern Recognition (ICPR).

[17] Cu V.L., Burie J.C., Ogier J.M., Liu C.L. (2019). A robust data hiding scheme using generated content for securing genuine documents. Proceedings of the 2019 International Conference on Document Analysis and Recognition (ICDAR).

[18] Wu M., Liu B. (2004). Data hiding in binary image for authentication and annotation. IEEE Trans. Multimed..

[19] Qi W.f., Li X.l., Yang B., Cheng D. (2008). Document watermarking scheme for information tracking. J. Commun..

[20] Hou Q., Junping D., Li L., Lu J., Chang C.C. (2015). Scanned binary image watermarking based on additive model and sampling. Multimed. Tools Appl..

[21] Tan L., Hu K., Zhou X., Chen R., Jiang W. (2019). Print-scan invariant text image watermarking for hardcopy document authentication. Multimed. Tools Appl..

[22] Yang X., Zhang W., Fang H., Ma Z., Yu N. (2023). Language universal font watermarking with multiple cross-media robustness. Signal Process..

[23] He B., Wu Y., Kang K., Guo W. (2009). A robust binary text digital watermarking algorithm for print-scan process. Proceedings of the 2009 WRI World Congress on Computer Science and Information Engineering.

[24] Li R.J., Chang L.W. (2005). Data hiding in binary images for annotation by parity check. Proceedings of the 2006 International Symposium on Intelligent Signal Processing and Communications.

[25] Yang H., Kot A.C. (2007). Pattern-based data hiding for binary image authentication by connectivity-preserving. IEEE Trans. Multimed..

[26] Wang C.C., Chang Y.F., Chang C.C., Jan J.K., Lin C.C. (2014). A high capacity data hiding scheme for binary images based on block patterns. J. Syst. Softw..

[27] Yang H., Kot A.C., Rahardja S. (2008). Orthogonal data embedding for binary images in morphological transform domain—A high-capacity approach. IEEE Trans. Multimed..

[28] Borges P.V.K., Mayer J. (2007). Text luminance modulation for hardcopy watermarking. Signal Process..

[29] Villán R., Voloshynovskiy S., Koval O., Vila J., Topak E., Deguillaume F., Rytsar Y., Pun T. (2006). Text data-hiding for digital and printed documents: Theoretical and practical considerations. Proceedings of the Security, Steganography, and Watermarking of Multimedia Contents VIII.

[30] Borges P.V.K., Mayer J., Izquierdo E. (2008). Robust and transparent color modulation for text data hiding. IEEE Trans. Multimed..

[31] Tan L., Sun X., Sun G. (2012). Print-Scan Resilient Text Image Watermarking Based on Stroke Direction Modulation for Chinese Document Authentication. Radioengineering.

[32] Amano T., Misaki D. (1999). A feature calibration method for watermarking of document images. Proceedings of the Fifth International Conference on Document Analysis and Recognition. ICDAR’99 (Cat. No. PR00318).

[33] Kim Y.W., Oh I.S. (2004). Watermarking text document images using edge direction histograms. Pattern Recognit. Lett..

[34] Xiao C., Zhang C., Zheng C. (2018). Fontcode: Embedding information in text documents using glyph perturbation. ACM Trans. Graph. (TOG).

[35] Qi W., Guo W., Zhang T., Liu Y., Guo Z., Fang X. (2019). Robust authentication for paper-based text documents based on text watermarking technology. Math. Biosci. Eng..

[36] Yao Y., Wang C., Wang H., Wang K., Ren Y., Meng W. (2024). Embedding secret message in Chinese characters via glyph perturbation and style transfer. IEEE Trans. Inf. Forensics Secur..

[37] Wang H., Zuo Q., Cao X., Zhao S., Li H. (2022). An anti-printing scanning watermarking algorithm based on fusion fonts. Advances in Artificial Intelligence and Security.

[38] Qi W., Liu Y., Guo W. (2014). An automatic Chinese font library generation method by modifying vector contour curves. Proceedings of the 2014 Tenth International Conference on Intelligent Information Hiding and Multimedia Signal Processing.

[39] Yang X., Zhang J., Fang H., Liu C., Ma Z., Zhang W., Yu N. (2023). AutoStegaFont: Synthesizing vector fonts for hiding information in documents. Proc. AAAI Conf. Artif. Intell..

[40] Yang X., Zhang J., Liu C., Fang H., Ma Z., Chen K., Zhang W., Yu N. (2025). Synthesizing Glyph Vectors for Practical Information Hiding in Documents. IEEE Trans. Dependable Secur. Comput..

[41] Sun N., Zhao C., Xie S., Ling H. (2024). InvertedFontNet: Font Watermarking based on Perturbing Style Manifold. Proceedings of the ICASSP 2024—2024 IEEE International Conference on Acoustics, Speech and Signal Processing (ICASSP).

[42] He C., Wu D., Zhang X., Wu H. (2024). Watermarking text documents with watermarked fonts. Proceedings of the 2024 ACM Workshop on Information Hiding and Multimedia Security.

[43] Wong K., Zhou J., Li K., Si Y.W., Wu X., Zhou J. (2025). FontGuard: A Robust Font Watermarking Approach Leveraging Deep Font Knowledge. IEEE Trans. Multimed..

[44] Brassil J.T., Low S., Maxemchuk N.F., O’Gorman L. (1995). Electronic marking and identification techniques to discourage document copying. IEEE J. Sel. Areas Commun..

[45] Maxemchuk N.F. (1994). Electronic document distribution. AT&T Tech. J..

[46] Low S.H., Maxemchuk N.F. (1998). Performance comparison of two text marking methods. IEEE J. Sel. Areas Commun..

[47] Huang D., Yan H. (2001). Interword distance changes represented by sine waves for watermarking text images. IEEE Trans. Circuits Syst. Video Technol..

[48] Alattar A.M., Alattar O.M. (2004). Watermarking electronic text documents containing justified paragraphs and irregular line spacing. Proceedings of the Security, Steganography, and Watermarking of Multimedia Contents VI.

[49] Zou D., Shi Y.Q. (2005). Formatted text document data hiding robust to printing, copying and scanning. Proceedings of the 2005 IEEE International Symposium on Circuits and Systems (ISCAS).

[50] Culnane C., Treharne H., Ho A.T. (2006). A new multi-set modulation technique for increasing hiding capacity of binary watermark for print and scan processes. Digital Watermarking.

[51] Xia Z., Wang S., Sun X., Wang J. (2013). Print-scan resilient watermarking for the Chinese text image. Int. J. Grid Distrib. Comput.

[52] Alotaibi R.A., Elrefaei L.A. (2018). Improved capacity Arabic text watermarking methods based on open word space. J. King Saud Univ.-Comput. Inf. Sci..

[53] Li L., Zhang H.J., Meng J.L., Lu Z.M. (2023). Robust PDF Watermarking against Print–Scan Attack. Sensors.

[54] Tseng Y.C., Pan H.K. (2001). Secure and invisible data hiding in 2-color images. Proceedings of the IEEE INFOCOM 2001. Conference on Computer Communications. Twentieth Annual Joint Conference of the IEEE Computer and Communications Society (Cat. No. 01CH37213).

[55] Varna A.L., Rane S V.A. Data hiding in hard-copy text documents robust to print, scan and photocopy operations. Proceedings of the IEEE International Conference on Acoustics, Speech and Signal Processing (ICASSP).

[56] Tancik M., Mildenhall B., Ng R. StegaStamp: Invisible Hyperlinks in Physical Photographs. Proceedings of the IEEE/CVF Conference on Computer Vision and Pattern Recognition (CVPR).

[57] Campbell N.D., Kautz J. (2014). Learning a manifold of fonts. ACM Trans. Graph. (ToG).

[58] Kaur A., Dong G., Basu A. (2022). GradXcepUNet: Explainable AI based medical image segmentation. Smart Multimedia.

